# Mitochondrial Dysfunction as Substrate for Arrhythmogenic Cardiomyopathy: A Search for New Disease Mechanisms

**DOI:** 10.3389/fphys.2019.01496

**Published:** 2019-12-10

**Authors:** Chantal J. M. van Opbergen, Lyanne den Braven, Mario Delmar, Toon A. B. van Veen

**Affiliations:** ^1^Department of Medical Physiology, Division of Heart & Lungs, University Medical Center Utrecht, Utrecht, Netherlands; ^2^Division of Cardiology, NYU School of Medicine, New York, NY, United States

**Keywords:** arrhythmogenic cardiomyopathy, cardiac metabolism, mitochondria, ATP, oxidative stress, plakophillin-2, calcium handling, connexin 43

## Abstract

Arrhythmogenic cardiomyopathy (ACM) is a familial heart disease, associated with ventricular arrhythmias, fibrofatty replacement of the myocardial mass and an increased risk of sudden cardiac death (SCD). Malignant ventricular arrhythmias and SCD largely occur in the pre-clinical phase of the disease, before overt structural changes occur. To prevent or interfere with ACM disease progression, more insight in mechanisms related to electrical instability are needed. Currently, numerous studies are focused on the link between cardiac arrhythmias and metabolic disease. In line with that, a potential role of mitochondrial dysfunction in ACM pathology is unclear and mitochondrial biology in the ACM heart remains understudied. In this review, we explore mitochondrial dysfunction in relation to arrhythmogenesis, and postulate a link to typical hallmarks of ACM. Mitochondrial dysfunction depletes adenosine triphosphate (ATP) production and increases levels of reactive oxygen species in the heart. Both metabolic changes affect cardiac ion channel gating, electrical conduction, intracellular calcium handling, and fibrosis formation; all well-known aspects of ACM pathophysiology. ATP-mediated structural remodeling, apoptosis, and mitochondria-related alterations have already been shown in models of PKP2 dysfunction. Yet, the limited amount of experimental evidence in ACM models makes it difficult to determine whether mitochondrial dysfunction indeed precedes and/or accompanies ACM pathogenesis. Nevertheless, current experimental ACM models can be very useful in unraveling ACM-related mitochondrial biology and in testing potential therapeutic interventions.

## Introduction

Arrhythmogenic cardiomyopathy (ACM) is a familial heart disease, associated with ventricular arrhythmias and an increased risk of sudden cardiac death (SCD). SCD often occurs as the first clinical manifestation, in particular in athletes and young adults (Basso et al., 2012). The prevalence of ACM in the general population ranges from 1:2000 to 1:5000, meaning that this disease is listed among the rare cardiovascular diseases (Basso et al., 2018). Age at time of diagnosis varies widely, with most patients being first diagnosed between 20 and 50 years of age (Bennett et al., 2019). ACM is characterized by progressive replacement of the myocardium with fatty and fibrous tissue, ending in impairment of the ventricular systolic function and ventricular dilatation (Corrado et al., 2017a). Cardiac remodeling in ACM patients is of right ventricular predominance, though left-ventricular or bi-ventricular involvement has been recognized (Bennett et al., 2019). The fibrofatty substitution likely contributes to the development of ventricular arrhythmias by creating an anatomic substrate (Corrado et al., 2017b) though cellular mechanisms of initiation have been invoked. ACM is considered an inheritable disease, as approximately 60% of the patients carry a genetic mutation, though likely not all genetic mutations related to ACM have been identified yet (Moncayo-Arlandi and Brugada, 2017). ACM is commonly considered as a disease of the intercalated disc (ID), whereby mutations in *Plakophilin-2* (*PKP2*) associate with most cases of ACM in which a genetic cause can be found. Mutations in other genes that encode desmosomal proteins [Desmocollin2 (DSC2), Desmoglein2 (DSG2), Plakoglobin (PKG), Desmoplakin (DSP)], have been linked to ACM as well (Basso et al., 2018). Desmosomes mediate cell–cell mechanical coupling of adjacent cardiomyocytes at the ID. ID proteins are not just considered “junctional” and “non-junctional” single function entities, but multitasking molecular complexes that jointly regulate electrical conduction and mechanical force (Leo-Macias et al., 2016a, b). Recent studies show that ID proteins are also involved in modulation of transcription pathways fundamental for homeostasis within cardiomyocytes. Specifically, the transcription of genes involved in the intracellular calcium (Ca^2+^) homeostasis can be modified by the expression of PKP2 (Cerrone et al., 2017; Montnach et al., 2018). Besides that, PKP2 loss provokes non-transcriptional related Ca^2+^ handling dysregulation, via ID-located Cx43 hemichannels and a change in the phosphorylation state of the Ryanodine receptor (RyR2) (Kim et al., 2019). This stands along the notion that PKP2, like many others in the cardiac myocyte, is a pleiotropic gene (Cerrone et al., 2019), and it highlights the fact that ACM is not only a desmosomal disease, but aberrant activation of intracellular signaling pathways and subsequent destabilization of the intracellular homeostasis likely provokes the structural and electrical changes in the heart. Besides desmosomal mutations, ACM can also be caused by mutations in genes directly involved in cardiac Ca^2+^ dynamics, such as the ryanodine receptor (RyR2) and phospholamban (PLN). Variants in RyR2 are classically linked to the inherited arrhythmogenic disease Catecholaminergic polymorphic ventricular tachycardia (CPVT), although RyR2 missense mutations have also been found in ACM patients (Tiso et al., 2001). Moreover, the Dutch founder mutation PLN-R14del has been associated with the development of ACM (Van Der Zwaag et al., 2013).

Sudden cardiac death in ACM patients often occurs in the subclinical phase of the disease, before structural changes are present. To prevent or interfere with ACM disease progression, more insight in the mechanisms related to electrical instability in ACM hearts is needed. At present, there is widespread interest on the link between cardiac arrhythmias and metabolic disease. A common focal point is the case of Diabetes Mellitus (DM) where a changed metabolic state and altered mitochondrial balance caused by elevated blood glucose levels, glucose fluctuation, and hypoglycemia can set the stage for cardiac arrhythmias (Grisanti, 2018). The altered energy metabolism and increased oxidative stress in DM cardiomyocytes have proven to influence intracellular Ca^2+^ handling, activate Ca^2+^/calmodulin-dependent protein kinase II (CaMKII), and cause mitochondria-induced cell death and fibrosis (El Hadi et al., 2019). Apoptosis and necrosis are important elements in structural remodeling of ACM hearts and mitochondria-related alterations have been shown in iPSC-derived cardiomyocytes (iPSC-CMs) from a patient with mutated PKP2 (Nishikawa et al., 1999; Caspi et al., 2013; Kim et al., 2013; Austin et al., 2019). Mitochondria are essential for providing adenosine triphosphate (ATP) to the cell and satisfying the energy demand for electrical activity and contraction (Yang et al., 2014). Mitochondria are also involved in the intracellular Ca^2+^ homeostasis of cardiomyocytes and ATP generated by the mitochondria fuels various ion pumps (Doenst et al., 2013; Kolwicz et al., 2013). Mitochondrial dysfunction can thereby affect the electrical stability of cardiomyocytes and favor arrhythmogenesis. In the specific case of ACM, this hypothesis has not been tested yet, though it is tempting to speculate that metabolic and mitochondrial dysfunction can serve as substrates for electrical and structural changes in ACM patients. Especially since previous studies have indicated differences in mitochondrial metabolism between the left ventricle (LV) and right ventricle (RV) in the heart and ACM is regarded a (right) chamber specific disease (Schlüter et al., 2018). In this review, we describe the mitochondrial (patho)physiology, the link between mitochondrial dysfunction and development of cardiac arrhythmias and explore a potential relation between mitochondrial dysfunction and typical hallmarks of ACM.

## Cardiac Mitochondrial (Patho)Physiology

The mechanical force required for cardiac contraction consumes large amounts of energy, which needs to be replenished via the mitochondria. The heart is capable of utilizing all classes of energy substrates for ATP production, including carbohydrates, lipids, amino acids, and ketone bodies (Kolwicz et al., 2013). Lactate and glucose are the major sources for energy production during the fetal stage. After birth, the heart undergoes a metabolic adaptation, switching substrate oxidation from glucose to fatty acids (FA). β-oxidation of free FA and oxidative phosphorylation (OXPHOS) become the primary mechanisms for ATP production and produces approximately 70% of all ATP in the heart (Torrealba et al., 2017). In the healthy heart, ATP production via OXPHOS is in balance with the rate of ATP hydrolysis. This balance mediates a constant ATP level in the cell, even during intense exercise (Stanley et al., 2005). The importance of mitochondrial function in cardiomyocytes is highlighted by their high abundance in the cell. Mitochondria occupy roughly 33% of the cellular volume in each ventricular cardiomyocyte and generate more than 95% of ATP consumed by the heart (Williams et al., 2015). ATP production is established via the mitochondrial membrane potential (ΔΨm = −180 mV). The ΔΨm creates a proton motive force which provides energy necessary to phosphorylate adenosine diphosphate (ADP) to ATP (Gambardella et al., 2017). This mechanism is also one of the main sources for reactive oxygen species (ROS) in the cell (Yang et al., 2014). Under physiological conditions, ΔΨm is tightly controlled and ATP production is in equilibrium with the energy demands of the cell. In response to pathological stimuli, such as ischemia or structural injury, alterations in ΔΨm diminish the cellular ATP level and increase ROS production (Gambardella et al., 2017). If ROS production exceeds the detoxifying (scavenging) capacity of the cell, it creates oxidative stress (Gambardella et al., 2017).

The two most important factors regulating the cardiac energy production are cellular concentrations of ADP and Ca^2+^ (Granatiero et al., 2017). Cytosolic Ca^2+^ levels tightly regulate enzymes of the tricarboxylic acid cycle (TCA) cycle and excitation-contraction coupling in the heart, thereby linking myocyte contraction and energy production (Granatiero et al., 2017). The heart uses about 60–70% of all generated ATP to facilitate contraction, the remaining 30–40% is used for control of various ion pumps, such as the sarco/endoplasmic reticulum Ca^2+^-ATPase (SERCA2a) (Doenst et al., 2013). About 15% of the cardiac energy is consumed via SERCA-ATPase activity, which highlights the energy cost of active Ca^2+^ signaling in the heart (Granatiero et al., 2017). The cellular Ca^2+^ homeostasis is maintained by both SR and mitochondrial Ca^2+^ cycling (Gadicherla et al., 2017). Mitochondria are defined by two structurally and functionally different membranes, the outer membrane (OMM) and the inner membrane (IMM) which surround the intermembrane space (IMS). The mitochondrial membrane contains large amounts of invaginations enclosing the mitochondrial matrix, referred to as “cristae.” Cristae junctions are tubular structures, which regulate and limit the random diffusion of molecules into the IMS. Mitochondria are linked to several other intracellular structures, especially the SR, enabling Ca^2+^ and lipid transfer between these two organelles. The mitochondrial matrix [Ca^2+^] is tightly regulated via mitochondrial Ca^2+^ influx and efflux. Ca^2+^ influx requires a balanced mitochondrial membrane potential (negative charge inside) and an electrogenic Ca^2+^ uniporter (Granatiero et al., 2017). Ca^2+^ crosses the outer mitochondrial membrane (OMM) through voltage dependent anion channels (VDAC), large channels that are permanently permeable to Ca^2+^. VDAC permits Ca^2+^ flux into the intermembrane space and the mitochondrial Ca^2+^ uniporter (MCU) allows Ca^2+^ entry from the intermembrane space into the mitochondrial matrix. The mitochondrial [Ca^2+^] plays an important role in regulation of mitochondrial ATP production (Williams et al., 2015; De Stefani et al., 2016). Ca^2+^ that enters the mitochondrial matrix can be pumped back into the intermembrane space via the mitochondrial Na^+^/Ca^2+^ exchanger (mNCX) and via ubiquitous H^+^/Ca^2+^ exchange. This means that the mitochondrial [Ca^2+^] is set by the dynamic balance between MCU Ca^2+^ entry and Ca^2+^ extrusion via the mNCX (Williams et al., 2015). Under physiological circumstances, the mitochondrial Ca^2+^ influx and efflux is relatively small and unlikely to affect the cytosolic Ca^2+^ concentration (Williams et al., 2015). However, a prolonged and sustained increase of the mitochondrial Ca^2+^ level opens the permeability transition pore (mPTP), collapses the membrane potential (ΔΨm) and activates apoptotic pathways (De Stefani et al., 2016).

Under pathological conditions, the fuel metabolism of the heart shifts back from FA oxidation (FAO) to an increased reliance on glucose, like in the fetal heart (Doenst et al., 2010, 2013). In addition, the metabolic gene expression shifts back to a fetal profile and the overall oxidative metabolism and energy reserve is thus reduced (Tuomainen and Tavi, 2017). Increased glucose consumption is characterized by enhanced glucose uptake and glycolysis, without prominent adaptations in glucose oxidation. As a consequence, glucose uptake and glucose oxidation become outbalanced. In combination with decreased FAO, the mitochondrial oxidative metabolism capacity gets depleted and ultimately the cardiac energy provision shrinks (Hajri et al., 2001). Cardiac hypertrophy and heart failure also enhance ketone body oxidation and disturb amino acid metabolism, particularly the branched-chain amino acid (BCAA) catabolism (Tuomainen and Tavi, 2017). The exact metabolic consequences and functional relevance of these changes remain to be understood.

## Mitochondrial-Associated Cardiac Arrhythmias

Cardiomyocyte excitability and electrical cell–cell coupling are critical factors for electrical activation and a synchronized contraction of the heart. Changes in ion channel composition, function, and localization increase the susceptibility toward arrhythmias (Marbán, 2002). Multiple ion channels undergo structural and functional remodeling driven by pathophysiological stimuli, such as generated by mitochondrial dysfunction (Marbán, 2002). In this chapter, we will outline the effects of mitochondrial dysfunction on parameters critical for cardiomyocyte excitability, electrical impulse propagation and contractility, likely predisposing to cardiac arrhythmias.

### ATP Driven Arrhythmogenic Substrate

**FIGURE 1:**
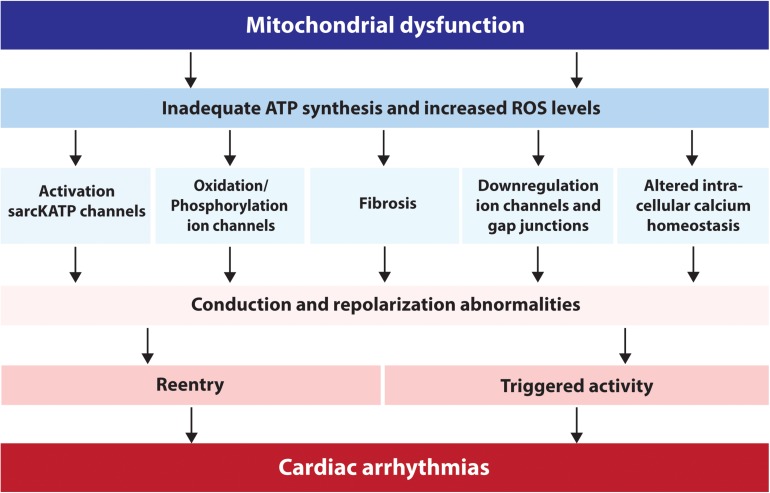
Schematic overview of the factors via which mitochondrial dysfunction can lead to cardiac arrhythmias. ATP, adenosine triphosphate; sarcKATP, ATP sensitive potassium channels on the sarcolemma; ROS, reactive oxygen species.

During mitochondrial ATP production, mitochondrial proteins, and enzymes are subjected to various post-translational modifications, such as phosphorylation and acetylation. These modifications can alter the activity of metabolic enzymes and affect downstream metabolic pathways (Parihar and Parihar, 2017). Enzymes important in FA metabolism, TCA cycle metabolism, electron transport chain (ETC) and OXPHOS can severely be dysregulated by an imbalanced ATP production (Parihar and Parihar, 2017). Dysfunction of the mitochondrial oxygen consuming capacity leads to destabilization of ΔΨm, which causes diminished ATP production and eventually energy deficiency in the cardiomyocyte (Parihar and Parihar, 2017). The impact of mitochondria on cellular excitability is mainly mediated by energy sensing, ATP sensitive K^+^ channels on the sarcolemma (sarcKATP) (Gambardella et al., 2017). These channels are negatively regulated via intracellular ATP levels and form a crucial link between metabolism and electrical function of the cardiomyocyte. An altered intracellular ATP/ADP ratio, as a consequence of mitochondrial dysfunction, results in opening of sarcKATP channels. This increases the K^+^ efflux and shortens the action potential duration (APD) (Gambardella et al., 2017). Shortening of the APD via sarcKATP channels reduces the time for Ca^2+^ influx via L-type Ca^2+^ channels (LTCC) during the action potential plateau phase and increases the time for Ca^2+^ extrusion via NCX (Nakaya, 2014). As a consequence, less intracellular Ca^2+^ is available and excitation-contraction coupling of cardiomyocytes is impaired (Gambardella et al., 2017). Via this mechanism, insufficient ATP levels in the cardiomyocyte diminish the contractile capacity of the cell. If a large amount of sarcKATP channels open at the same time, they hold the membrane potential very close to the K^+^ Nernst equilibrium potential and keep the cardiomyocytes in a non-excitable state (Brown and O’Rourke, 2010). As consequence, in regions where sarcKATP channels are open due to metabolic stress, a ‘sink’ is created for depolarization of the myocardium. This phenomenon is called the ‘metabolic sink.’ It impairs electrical conduction and is suggested to be an important substrate for cardiac arrhythmias (Figure 1) (Brown and O’Rourke, 2010). Blocking sarcKATP channels with HMR1883 showed to reduce the incidence of ventricular arrhythmia in rats, rabbits, pigs, and dogs (Billman et al., 1998; Wirth et al., 1999; Fischbach et al., 2004; Vajda et al., 2007). These findings have been confirmed in clinical studies where sarcKATP channel blockers reduced the incidence of ventricular arrhythmias in patients with decompensated heart failure and DM (Cacciapuoti et al., 1991; Aronson et al., 2003).

### Oxidative Stress Related Arrhythmogenic Substrate

A different and important aspect of mitochondrial dysfunction is the production of ROS, which may result in oxidative stress (Gambardella et al., 2017). Oxidative stress is defined as the imbalance between the generation of ROS and the cellular antioxidant defense (El Hadi et al., 2019). ROS are unstable molecular structures such as superoxide (${\text{O}}_{2}^{∙-}$), hydrogen peroxide (H_2_O_2_), peroxynitrite (ONOO^–^) and hydroxyl radicals (OH). They can damage proteins and lipids within the cell, activate intracellular signaling cascades and induce cellular dysfunction or cell death (Tse et al., 2016; El Hadi et al., 2019). To protect cellular functions from ROS, cells have two defense mechanisms: enzymatic and non-enzymatic pathways. The enzymatic pathway includes superoxide dismutase, catalase, and glutathione peroxidase. Together they are responsible for breaking down superoxide into water and oxygen. The non-enzymatic pathways include a variety of redox-defense systems, for example antioxidant scavengers (Jeong et al., 2012). The arrhythmogenic effect of ROS depends on ROS-induced ROS release (RIRR). This is an autocatalytic process by which high levels of ROS induce further ROS release from mitochondria (Gambardella et al., 2017). Increased oxidative stress through ROS is associated with the abnormal function of intracellular organelles, including the SR and mitochondria (Tse et al., 2016). Via several pathways, oxidative stress leads to repolarization- and conduction abnormalities, eventually resulting in cardiac arrhythmias (Figure 1) (Tse et al., 2016). For example, destabilization of ΔΨm can cause oxidation of ion channels via ROS, and/or indirectly through phosphorylation of ion channels via activation of protein kinases (Figure 1) (Foteinou et al., 2015; Yang et al., 2015). Increased ROS production can also activate the unfolded protein response (UPR) that accordingly reduces ion channel functionality and creates repolarization abnormalities (Tse et al., 2016). In addition, oxidative stress impairs gap junction conduction and electrical coupling between cardiomyocytes. Elevation of ROS levels decreases the amount of Cx43 protein in the cell and hampers gap junction conduction, resulting in cardiac arrhythmias and SCD (Figure 1) (Iravanian et al., 2011; Sovari et al., 2011). Moreover, ROS production alters the intracellular Ca^2+^ homeostasis (by inducing SR Ca^2+^ sparks and increasing cytosolic Ca^2+^ levels) and causes fibrosis formation (Voigt et al., 2012; Köhler et al., 2014) creating two additional substrates for conduction abnormalities, triggered activity and re-entry based arrhythmias (Figure 1) (Köhler et al., 2014). The relationship between ROS production, Ca^2+^ handling disturbances and structural remodeling will be explained later in this review.

### Effect of Oxidative Stress on Cardiac Calcium Dynamics

#### Mitochondrial Calcium Overload and Oxidative Stress

**FIGURE 2:**
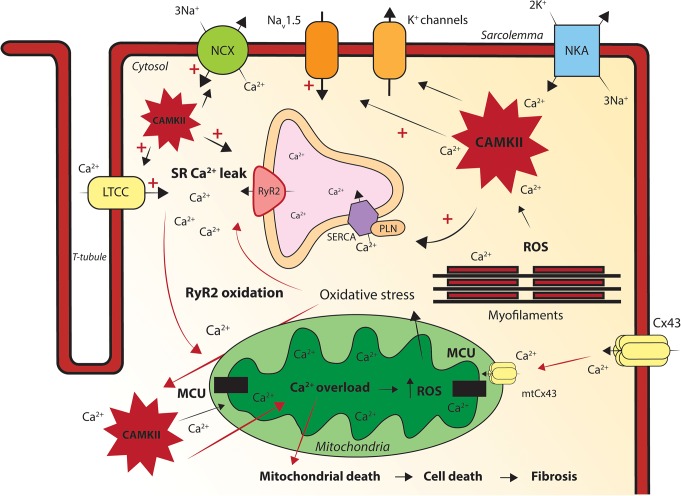
Graphic illustration of the processes via which mitochondrial dysfunction causes intracellular calcium handling disturbances. Oxidative stress induces mitochondrial calcium (Ca^2+^) overload via the MCU. Mitochondrial Ca^2+^ overload increases reactive oxygen species (ROS) production, oxidizes ryanodine receptors (RyR2) and will subsequently cause sarcoplasmic reticulum (SR) Ca^2+^ leakage. Excessive SR Ca^2+^ release creates elevated cytosolic Ca^2+^ levels and free Ca^2+^ ions will be transported into the mitochondria via the mitochondrial Ca^2+^ uniporter (MCU). The increased intracellular [Ca^2+^] activates the pathologic Ca^2+^ dependent protein Ca^2+^/calmodulin-dependent protein kinase II (CaMKII). ROS and Ca^2+^ induced sustained activation of CaMKII hyper-phosphorylate the l-type Ca^2+^ channel (LTCC), sodium channel (Na_V_1.5) and RyR2, increasing the sodium current (I_Na_), [Na]_i_ and [Ca^2+^]_I_. Connexin43 (Cx43) and mitochondrial Cx43 (mtCx43) can cause cytosolic and mitochondrial Ca^2+^ overload. Mitochondrial Ca^2+^ overload causes mitochondrial death, apoptosis and fibrosis in the heart. Disturbed intracellular Ca^2+^ dynamics and/or fibrosis formation will eventually lead to conduction and repolarization abnormalities in the heart, causing triggered activity and re-entry based cardiac arrhythmias. LTCC, l-type calcium channel; NCX, sodium calcium exchanger; NKA, sodium potassium ATPase; RyR2, ryanodine receptor; SERCA, sarco/endoplasmic reticulum Ca^2+^-ATPase; PLN, phospholamban; Cx43, connexin43; mtCx43, mitochondrial connexin 43; MCU, mitochondrial Ca^2+^ uniporter; SR, sarcoplasmic reticulum; CAMKII, Ca^2+^/calmodulin-dependent protein kinase II; ROS, reactive oxygen species.

The link between disturbed cytosolic Ca^2+^ levels and cardiac arrhythmias is well-established, although mitochondrial Ca^2+^ dysregulation as substrate for arrhythmias is less clear (Brown and O’Rourke, 2010). Oxidative stress can induce mitochondrial Ca^2+^ overload via the MCU. An overload of mitochondrial Ca^2+^ results in opening of mPTP, collapse of ΔΨm, release of cytochrome c and eventually myocyte death (Figure 2) (Gambardella et al., 2017). It has been demonstrated that the H_2_O_2_-induced activation of MCU and subsequent mitochondrial Ca^2+^ overload can be decreased by overexpression of microRNA 25 (MiR-25). Overexpression of MiR-25 reduced the MCU protein levels, diminished H_2_O_2_ driven elevation of the mitochondrial Ca^2+^ levels and inactivated the mitochondrial apoptotic pathway (Pan et al., 2015). Prevention of mitochondrial Ca^2+^ overload, via inhibition of MCU, has also proven protective against the development of cardiac arrhythmias (Hamilton et al., 2018). Mitochondrial Ca^2+^ overload promotes excessive ROS production and oxidative stress, of which the consequences have already been discussed in the previous paragraphs.

#### Ryanodine Receptor Oxidation by ROS

RyR2 is a crucial component of Ca^2+^-induced Ca^2+^-release (CICR) and highly sensitive to oxidative stress, as it is oxidized by increased ROS levels in the cytosol (Joseph et al., 2016; Hamilton et al., 2018). Oxidation of RyR2 causes opening of the channel pore and Ca^2+^ leak out of the SR. This SR Ca^2+^ leak results in Ca^2+^ sparks, cytosolic Ca^2+^ overload and SR Ca^2+^ depletion, inducing delayed after depolarization’s (DADs) and impairment of the cardiac contractile force (Figure 2) (Voigt et al., 2012; Santulli et al., 2015). Enhanced cytosolic Ca^2+^ levels cause mitochondrial Ca^2+^ overload via an increased MCU Ca^2+^ uptake, eventually provoking a pro-arrhythmic vicious cycle of malignant high cellular Ca^2+^ levels (Figure 2) (Voigt et al., 2012; Santulli et al., 2015). Elevated cytosolic Ca^2+^ levels also activate a variety of pathogenic Ca^2+^ sensitive signaling pathways in cardiomyocytes, as for example the one including Ca^2+/^Calmodulin-dependent kinase II (CaMKII) (van Opbergen et al., 2017).

#### Calcium/Calmodulin-Dependent Protein Kinase II Activation by ROS

CaMKII is a multifunctional serine/threonine kinase located at the cell membrane, cytoplasm and in the nucleus of cardiomyocytes. Ca^2+^ and Calmodulin facilitate CaMKII activation under physiological conditions, though CaMKII is also a substrate for its own monomers (Erickson, 2014). Under pathological conditions ROS can directly oxidize CaMKII, leading to its persistent activity (Foteinou et al., 2015). Once activated (via ROS or elevated cytosolic Ca^2+^ levels) CaMKII can target several ion channels and transcription factors, which coordinate cardiac electrical and mechanical mechanisms (Figure 2) (Rokita and Anderson, 2012). CaMKII can for example hyper-phosphorylate the L-type Ca^2+^ channel (Ca_V_1.2), Na_V_1.5 and RyR2, thereby increasing cytosolic Ca^2+^ levels (Rokita and Anderson, 2012). This leads to contractile dysfunction and after depolarization’s, as discussed before. Moreover, the increased cytosolic Ca^2+^ levels can lead to mitochondrial Ca^2+^ overload, further increasing ROS production and ultimately resulting in cell death (Figure 2) (Gambardella et al., 2017). It has been shown that oxidative CaMKII activation leads to after depolarization’s in isolated rabbit cardiomyocytes, caused by phosphorylation of L-type Ca^2+^ (LTCC) and Na^+^ channels (Yang et al., 2018). In addition, mitochondrial-targeted antioxidant treatment has shown to suppress early after depolarization’s (EADs) in an *in silico* model of guinea pig cardiomyocytes (Yang et al., 2018). Phosphorylation of Na_V_1.5 delays the I_Na_ recovery time after inactivation and enhances the persistent late Na^+^ current (Wagner et al., 2006). Under pathophysiological conditions, the CaMKII induced increased Na^+^ peak current further elevates diastolic Ca^2+^, as increased cytosolic Na^+^ levels stimulate the Ca^2+^ influx via the sodium- Ca^2+^ exchanger (NCX) (Yao et al., 2011). Elevated cytosolic Ca^2+^ levels enhance the phosphorylation and activation of CAMKII, ending in a positive feedback loop in which a CaMKII-dependent increase of I_CaL_, alters the Ca^2+^ homeostasis and persistent activation of CaMKII. When during the plateau phase more Ca^2+^ and Na^+^ enter the cell, EADs can develop, provoking ectopic activity in the heart (Wagner et al., 2006). Overexpression of CaMKIIδ in rabbit cardiomyocytes has proven to increase the I_Na_, [Na]_i_ and cause accumulation of Ca^2+^_i_ (Wagner et al., 2011). CaMKIIδ knock out in murine cardiomyocytes blunts the ROS (H_2_O_2_)-induced intracellular accumulation of Na^+^ and Ca^2+^ and its subsequent incidence of ventricular arrhythmias, hyper-contraction, and SCD (Wagner et al., 2011). This implies that ROS-induced CaMKII activation causes cellular Na^+^ overload and, as a consequence, disturbs the Ca^2+^ balance in the cell.

#### The Effect of ROS on the L-Type Calcium Channel and Na^+^/Ca^2+^ Exchanger

The L-type Ca^2+^ channel (LTCC) is a voltage gated Ca^2+^ channel that couples electrical activation, via an action potential, to contraction of the cardiomyocyte. The effects of ROS on LTCCs are controversial (Yang et al., 2014). On one hand, it has been shown that elevated mitochondrial ROS levels increase the I_Ca_ in guinea pig ventricular cardiomyocytes (Viola et al., 2007). On the other hand, increased ROS levels decreased I_Ca_ in hamster ventricular cardiomyocytes. This could be due to energy depletion or Ca^2+^ overload, rather than oxidation of the LTCC (Hammerschmidt and Wahn, 1998). It should be taken into account that results can vary between animal species and different types of ROS. The subset of ROS components differs in reactivity and oxidation potential (Yang et al., 2014). The Na^+^/Ca^2+^ exchanger (NCX) is an antiporter membrane protein which mainly works in the forward mode, pumping 3 Na^+^ ions into the cardiomyocyte in exchange for 1 Ca^2+^ ion (Amin et al., 2010). Whether NCX acts in the forward or

reversed mode depends on the driving force of the intracellular ion concentrations: high cytosolic [Ca^2+^] favors the forward mode, whereas high cytosolic [Na^+^] and a positive membrane potential favor the reverse mode (Driessen et al., 2014). The effect of oxidative stress on NCX activity is also controversial, as ROS has proven to both stimulate and decrease NCX activity (Zhang et al., 2016). Nevertheless, both RyR2 oxidation and CaMKII activation elevates the cytosolic [Ca^2+^], forcing NCX into the forward mode, inducing a depolarizing current and making the cell susceptible for DADs (Driessen et al., 2014).

To conclude, mitochondrial dysfunction can result in inadequate ATP synthesis and an increased ROS production. Insufficient supply of ATP to the cardiomyocytes results in activation of sarcKATP channels, which impair cardiomyocyte excitability, electrical impulse propagation and cardiac contractility. Furthermore, enhanced cellular ROS levels cause RIRR, aberrant opening of various ion channels, repolarization abnormalities, impaired electrical conduction and eventually provokes different types of malignant cardiac arrhythmias.

Oxidative stress resulting from mitochondrial dysfunction can induce mitochondrial Ca^2+^ overload via the MCU. Mitochondrial Ca^2+^ overload increases ROS production, oxidizes RyR2 and will subsequently cause SR Ca^2+^ leakage. Excessive SR Ca^2+^ release creates elevated cytosolic Ca^2+^ levels and free Ca^2+^ ions will be transported into the mitochondria via MCU. Mitochondrial Ca^2+^ reuptake and subsequent ROS production creates a vicious pro-arrhythmic environment in the cardiomyocyte. The increased intracellular [Ca^2+^] prolongs the APD, initiates after depolarization’s, causes contractile dysfunction and importantly activates pathologic Ca^2+^ dependent signaling pathways as for example CaMKII. ROS and Ca^2+^ induced sustained activation of CaMKII will hyper-phosphorylate Ca_V_1.2, Na_V_1.5 and RyR2, increasing I_Na_, [Na]_i_ and [Ca^2+^]_i__._ Via these mechanism, a separate vicious pro-arrhythmic cycle of altered Ca^2+^ homeostasis and CaMKII activation is initiated in the cell (Wagner et al., 2011; Bertero and Maack, 2018). Whether these mechanisms, all or in part, are present in ACM hearts, remains to be studied.

## Mitochondrial Dysfunction and Acm

Arrhythmogenic cardiomyopathy is a familial heart disease, associated with ventricular arrhythmias and progressive fibrofatty replacement of the myocardium. Ventricular arrhythmias in individuals with ACM are usually exercise-related and range from frequent premature ventricular complexes (PVCs) to ventricular tachycardia (VT) and ventricular fibrillation (VF) (Benito et al., 2011). The intimate mechanism of these ventricular arrhythmias is still unclear but triggered activity and reentry-based conduction are two likely factors. These two pro-arrhythmic factors are mainly based on disturbed intercellular communication and intracellular Ca^2+^ dynamics, fibrotic infiltrates in the heart and ion channel dysfunction (Cerrone et al., 2017; van Opbergen et al., 2017; Kim et al., 2019). Examination of cardiac biopsies and post-mortem cardiac material revealed diminished expression of Cx43 and Na_V_1.5 at the ID of ACM patients, two critical determinants of cardiomyocyte excitability and electrical cell–cell coupling (Noorman et al., 2013). In this chapter, we will explore a potential link between parameters known to be involved in ACM development and mitochondrial dysfunction.

### Mitochondrial Cx43 Hemichannels and Disturbed Calcium Dynamics in ACM

Gap junctions regulate cell–cell coupling between cardiomyocytes, conducting the passage of small molecules, metabolic substrates and electrical current between the adjacent cells (Kar et al., 2012). In ACM patients, a diminished expression of Cx43 at the ID was demonstrated in a large cohort of patients (Basso et al., 2018). Under pathological conditions, Cx43 can also be redistributed as Cx43 hemichannels to the lateral side of the cardiomyocyte (Cogliati et al., 2016). Cx43 hemichannels are large non-selective conduction pores, transporting small molecules and ions via concentration gradients across the cellular membrane. By this mechanism, hemichannels can release ATP, exchange Na^+^ and Ca^2+^ ions and release K^+^ ions (Boengler and Schulz, 2017). Reduced ID Cx43 expression and lateral Cx43 localization contribute to abnormal electrical impulse propagation and thereby create an arrhythmogenic substrate in the ACM heart. In addition, Cx43 hemichannels in the perinexus of the cardiomyocyte (outside of the gap junctions) are suggested as important arrhythmogenic substrates in a mouse model of PKP2 deficiency (Kim et al., 2019).

Cx43 resides not only in the ID, but is also present on the mitochondrial membrane, though almost exclusively at mitochondria that are located directly beneath the sarcolemma (Boengler and Schulz, 2017). It has been suggested that ROS can increase the expression of mtCx43, via activation of the p38-MAPK pathway (Matsuyama and Kawahara, 2011). Recent studies uncovered the contribution of mtCx43 hemichannels to mitochondrial Ca^2+^ entry (Gadicherla et al., 2017). Cardiac mitochondria play an important role in the intracellular ion homeostasis of cardiomyocytes, especially for Ca^2+^ ions (Kolwicz et al., 2013). Under pathological circumstances, mitochondrial Ca^2+^ overload increases ROS production, induces oxidative stress and thereby interferes with the SR Ca^2+^ cycling and elevates cytosolic Ca^2+^ levels (Gambardella et al., 2017). Close proximity of sarcolemmal Cx43 hemichannels, mitochondria and mtCx43 likely facilitate ATP and Ca^2+^ exchange that under pathological conditions can interfere with intracellular and mitochondrial Ca^2+^ dynamics. Elevated cytosolic Ca^2+^ levels activate several pro-arrhythmic pathways and cause triggered activity in the heart (Katra and Laurita, 2005; van Opbergen et al., 2017).

Nowadays, it is more and more recognized that in ACM patients RV mechanical deterioration precedes, or occurs in parallel, with the electrical disease progression, potentially initiated via Ca^2+^ handling disturbances and/or structural remodeling (Mast et al., 2016; Cerrone et al., 2017; Taha et al., 2019). In murine PKP2cKO-RV cardiomyocytes, Cx43 hemichannels in the sarcolemma increase the membrane permeability and cause intracellular Ca^2+^ overload and ISO-induced ventricular arrhythmias (Kim et al., 2019). It is tempting to speculate that an increased mitochondrial membrane permeability, via mtCx43 hemichannels, allows excessive entry of Ca^2+^ into the mitochondria and induces mitochondrial Ca^2+^ overload upon loss of PKP2 expression (Figure 2). As well, previous studies have suggested that mitochondrial metabolism differs between the LV and RV. Therefore, it might be the case that mitochondrial dysfunction alters intracellular Ca^2+^ sensitive pathways and break the Ca^2+^ balance in the cell, leading to RV initiated electrical disturbances and structural remodeling in ACM patients. This could for example be mediated via reduced ATP production, enhanced ROS production, direct interference with the Ca^2+^ handling related proteins or mtCx43 hemichannels, as explained in the previous chapter (Figure 2).

### Mitochondrial Related Fibrosis Formation in ACM

Besides ventricular arrhythmias is ACM characterized by fibrofatty replacement of the ventricular myocardium (Bennett et al., 2019). Fibroblasts can become activated upon injury or environmental stress, triggering their differentiation into myofibroblasts, the primary collagen-producing cell (Nguyen et al., 2014). Myofibroblast differentiation further induces pro-fibrotic responses, including the production of extracellular matrix proteins, collagen and cytokines (Thannickal et al., 2003). Excessive deposition of collagen, in between or in place of the healthy myocardium, interferes with the cardiac electrophysiology. Collagen deposition in the heart acts as a physical barrier to conduction, facilitating re-entry (Nguyen et al., 2017). Molecular signatures underlying the epicardium-initiated fibrofatty replacement in ACM hearts remain largely unexplored (Corrado et al., 2017b; Sepehrkhouy et al., 2017). Previous studies have proposed that abnormal trafficking of intracellular proteins to the ID and subsequent Wnt/β-catenin and Hippo-YAP pathway activation underlie the disease pathogenesis in ACM (Pilichou et al., 2016). Involvement of mitochondrial related processes in fibrofatty infiltration of ACM hearts have not been well-studied and may be an interesting new target. Especially, since previous studies showed apoptosis in ACM-affected hearts and RV vs. LV differences in (mitochondrial) Ca^2+^ handling during the concealed stage of the disease (Nishikawa et al., 1999; Akdis et al., 2016; Austin et al., 2019; Kim et al., 2019). This suggests that alterations in mitochondrial function might serve as early component in ACM, preceding overt structural disease and apoptosis.

#### ATP- and ROS-Induced Collagen Production in ACM

**FIGURE 3:**
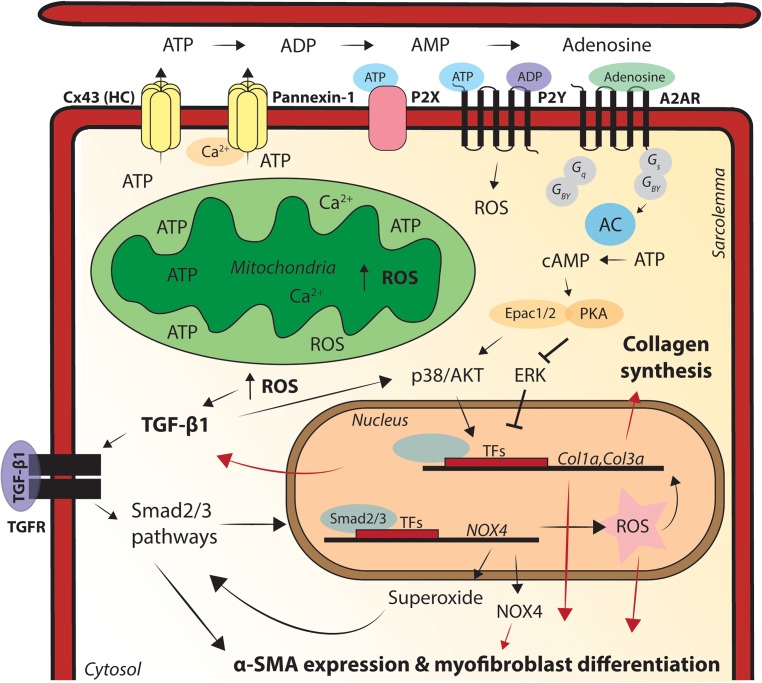
Graphic illustration of the processes via which mitochondrial dysfunction causes pro-fibrotic remodeling in the heart. Relocation of connexin43 (Cx43) hemichannels to the lateral membrane enables release of adenosine triphosphate (ATP) into the extracellular space, via mitochondria close residing at sides of cell–cell contact. ATP can directly activate P2X/Y channels and also convert into adenosine diphosphate (ADP), adenosine monophosphate (AMP) and adenosine. Adenosine subsequently activates pro-fibrotic signaling pathways, via adenosine 2A receptor (A2AR) binding. Pannexin-1 is activated via enhanced cytosolic calcium (Ca^2+^) levels and sarcoplasmic (SR) Ca^2+^ release and has the same ATP-mediated pro-fibrotic factor capacity as Cx43 hemichannels. Superoxide activates transforming growth factor (TGF)-β1 expression, fibroblast differentiation into myofibroblasts and subsequent extracellular matrix (ECM) deposition. TGF-β1-mediates reactive oxygen species (ROS) production, regulates α-smooth muscle actin (SMA) expression in the cell and the conversion of fibroblasts into myofibroblasts and TGF-β1 promotes the production of superoxide via stimulating NADPH oxidase 4 (Nox4). Nox4 superoxide production is also regulated via Smad2/3 activation, Smad2/3 activation leads to enhanced activation of Nox4, creating a pro-fibrotic positive-feedback loop in the heart. Fibrosis formation in the heart acts as a physical barrier to conduction, facilitating re-entry based cardiac arrhythmias. Cx43, connexin43; ATP, adenosine triphosphate; ADP, adenosine diphosphate; AMP, adenosine monophosphate; A2AR, adenosine 2A receptor; AC, adenylate cyclase; cAMP, cyclic AMP; PKA, protein kinase A; ROS, reactive oxygen species; TFs, transcription factors; Col1α, collagen 1α; Col3α, collagen 3α; Nox4, NADPH oxidase 4; TGF-β1, transforming growth factor beta 1; α-SMA, alpha smooth muscle actin.

Multiple cellular and animal models have been used to explore the role of PKP2 in pro-fibrotic processes. Dubash et al. (2016) showed that paracrine pathways might be responsible for inducing fibrosis in the setting of PKP2 deficiency. In HL-1 cells lacking PKP2, transforming growth factor (TGF)-β1 expression was increased and the p38-mitogen-activated protein kinases (MAPK) pro-fibrotic program was activated. Importantly, this study also showed that p38-MAPK was activated in neighboring, PKP2-positive, HL-1 cells (Dubash et al., 2016). These data strongly suggest cell–cell communication between PKP2 positive and negative cells and the presence of a paracrine pathway for induction of the pro-fibrotic processes. The molecular carrier of this cell–cell communication was not identified. Recently, Cerrone et al. (2018) explored adenosine as a possible pro-fibrotic molecular cell–cell messenger in the PKP2-deficient heart. Previous studies showed that binding of adenosine to the adenosine 2A receptor (A2AR) enhances collagen deposition in skin, lung, and liver tissue (Shaikh and Cronstein, 2016). A2AR activation can induce TGF-β1 expression and activate GSK3-β and Wnt-signaling pathways (Giambelluca et al., 2013; Shaikh et al., 2016; Zhang et al., 2017). In the heart, ATP can rapidly convert to adenosine, which bind to its G-coupled protein receptor (A2AR) (Peng et al., 2008; Fernández et al., 2013). In the study of Cerrone et al. (2018), we showed that ATP release was significantly higher in PKP2-deficient cells and that this effect was blunted when Cx43 was also silenced. This suggests that Cx43 hemichannels may act as a conduit for ATP release in PKP2-deficient cells (Figure 3). As proposed in Kim et al. (2019), reduced intercellular adhesion strength by PKP2 deficiency may disrupt the gap junction plaque integrity and create a pool of Cx43 hemichannels at the ID (perinexus). Cx43 hemichannels can passively leak intercellular solutes as ATP, via mitochondria closely resembling at the site of cell–cell contact (Leo-Macías et al., 2015). In this particular study, Cerrone et al. (2018) treated PKP2cKO mice with Istradefylline, a specific A2AR antagonist and indeed less collagen was found in both ventricles of PKP2-cKO mice after treatment.

#### ROS-Induced Fibroblast Differentiation in ACM

Besides Cx43 hemichannels, pannexin-1 channels are also involved in cardiac fibrosis formation, likely via release of ATP from the cardiomyocytes (Nishida et al., 2008; Kar et al., 2012; Li et al., 2015). Pannexin-1 can be activated by Ca^2+^ released from the SR and allow passage of Ca^2+^, ATP and other small molecules across the membrane, in a manner similar to that of Cx43 hemichannels (Figure 3) (Li et al., 2015; Xu et al., 2018). Oxidative stress activates Pannexin-1 hemichannels and does increase ATP release of cardiomyocytes via Pannexin-1 (Zhang et al., 2008; Dolmatova et al., 2012). Pannexin-1 mediated ATP release activates G-coupled P2X and P2Y receptors, further triggering the accumulation of ROS, via their underlying pathways (Figure 3) (Onami et al., 2014; Díaz-Vegas et al., 2015; Xu et al., 2018). P2X and P2Y activation enhances the transcription of fibrotic genes such as TGF-β1 (Nishida et al., 2008). ATP not only directly activates P2X and P2Y receptors, but can also be converted to AMP and adenosine, activating A2AR and underlying pro-fibrotic pathways, mechanisms proven to be involved in fibrosis formation in PKP2-cKO mice (Figure 3) (Shaikh et al., 2016; Cerrone et al., 2018).

The fact that Pannexin-1 is activated by intracellular Ca^2+^ levels and SR Ca^2+^ release, both increased by ROS-induced RyR2 oxidation, highlights Pannexin-1 as an interesting new target in mitochondrial related pro-fibrotic processes of ACM. In addition to the indirect (Pannexin-1 mediated) ROS-induced fibrosis formation, it has been demonstrated that mitochondrial ROS also directly promotes fibroblast differentiation (Choi et al., 2014). For example, superoxide activates TGF-β1 expression, fibroblast differentiation into myofibroblasts and subsequent extracellular matrix (ECM) deposition (Cucoranu et al., 2005; Choi et al., 2014). Myofibroblasts are characterized by expression of contractile proteins, such as smooth muscle α-actin (α-SMA). Cucoranu et al. (2005) showed that TGF-β1-mediated ROS production regulates α-SMA expression in the cell and the conversion of fibroblasts into myofibroblasts. In human cardiac fibroblasts, TGF-β1 promotes the production of superoxide via stimulating NADPH oxidase 4 (Nox4) and superoxide subsequently stimulates α-SMA expression and ECM deposition. Nox4 superoxide production is regulated via Smad2/3 activation. Moreover, Smad2/3 activation leads to enhanced activation of Nox4, creating a pro-fibrotic positive-feedback loop (Figure 3) (Cucoranu et al., 2005).

The most direct link between mitochondrial-related processes and collagen production in ACM has been found in PKP2-cKO mice. However, there are several other ACM models were involvement of TGF-β1, GSK3-β, and Wnt-signaling pathways in cardiac fibrosis formation has been proven (Chelko et al., 2016; Calore et al., 2019; Zheng et al., 2019). If activation of these pathways indeed is preceded by increased ROS levels, ATP release, P2X, P2Y, and/or A2AR activation would be an interesting topic for upcoming studies.

### PPAR-Induced Apoptosis and Lipogenesis in ACM

Cardiac metabolic activity is tightly regulated by expression levels of metabolic enzymes and their post-translational modifiers. One important class of transcriptional regulators for FA use are nuclear Peroxisome proliferator–activated receptors (PPAR). PPARα is the predominant isoform in the heart, although PPARδ and PPARγ also regulate lipid metabolism (Kersten et al., 2000; Lopaschuk et al., 2010). PPARγ is mainly expressed in adipose tissue and contributes to adipogenesis, the differentiation of pre-adipocytes to adipocytes (Lecarpentier et al., 2010). PPARs interact with members of the PPAR-γ coactivator-1 (PGC-1) family and co-activation of these proteins increases the FA uptake capacity and FAO (Rowe et al., 2010). Recently, an interesting link was found between cardiomyocyte metabolism, apoptosis, and lipogenesis in an induced pluripotent stem cell cardiomyocyte (iPSC-CM) model of ACM. Dermal fibroblasts from patients harboring a *PKP2* mutation were reprogramed to generate iPSC-CMs and mutant PKP2 iPSC-CMs demonstrated exaggerated lipogenesis and apoptosis (Caspi et al., 2013; Kim et al., 2013). Kim et al. (2013) presented PPARα, β/δ, and γ as key regulators in these processes, especially PPARγ seem to be activated. Experimental PPARγ over-activation in PKP2-iPS-CMs also induced exaggerated lipogenesis and pronounced apoptosis, caused by pathogenic PPARγ and PPARα co-activation (Kim et al., 2013). In addition, Kim et al. (2013) assessed the involvement of ROS in pathogenesis of PKP2 iPSC-CM and decreased ROS levels could indeed prevent cardiomyocyte death of these iPSC-CM. As discussed earlier, the heart experiences a metabolic switch after birth, changing substrate oxidation from glucose to FA (Torrealba et al., 2017). After pathogenic co-activation of PPARγ and PPARα, the mutant PKP2 iPSC-CMs also demonstrated a fuel shift from FA and glucose metabolism to pre-dominant glucose utilization (Kim et al., 2013). These data strongly suggest that pathological metabolic changes underlie disease progression in ACM patients harboring a *PKP2* mutation. The engagement of PPARs in a PKP2-related cardiomyopathy has been confirmed in the PKP2-cKO mouse model (Cerrone et al., 2017). KEGG analysis of transcriptional regulation in PKP2-cKO murine hearts presented the adipocytokine signaling pathway as one of the most upregulated pathways, with a predominant effect on PPARα (Cerrone et al., 2017). Moreover, GTEx-based analysis of human RNA abundance in the heart revealed that PPARα is a member of a PKP2-centered gene network, thus suggesting a direct regulation of PPARα levels by PKP2 expression (Montnach et al., 2018).

In conclusion, ACM is a progressive and predominantly RV disease, characterized by ventricular arrhythmias and fibrofatty infiltrates in the myocardium. Mitochondrial interference with the Ca^2+^ homeostasis in the cell, directly or indirectly via Cx43 hemichannals and mtCx43, tentatively serves as an important substrate in RV initiated electrical disturbances and structural remodeling in ACM patients. In murine PKP2cKO-RV cardiomyocytes, Cx43 hemichannels in the sarcolemma increase the membrane permeability and cause intracellular Ca^2+^ overload and ISO-induced ventricular arrhythmias. ROS can increase the expression of mtCx43 and close the proximity of sarcolemmal Cx43 hemichannels, mitochondria and mtCx43 might facilitate ATP and Ca^2+^ exchange that under pathological conditions can interfere with intracellular and mitochondrial Ca^2+^ dynamics. In addition, cellular ATP release and increased oxidative stress are important pro-fibrotic aspects in the (ACM) heart. Cx43 hemichannels in the perinexus enable release of ATP into the extracellular space, via mitochondria close residing at sides of cell–cell contact. ATP can directly activate P2X/Y channels and also convert into ADP, AMP, and adenosine. Adenosine subsequently activates pro-fibrotic signaling pathways (as TGF-β1 activation) via A2AR binding. Pannexin-1 has the same ATP-mediated pro-fibrotic factor capacity as Cx43 hemichannels. Interestingly, pannexin hemichannels are activated via enhanced cytosolic Ca^2+^ levels and SR Ca^2+^ release, which is increased by ROS-induced RyR2 oxidation. Furthermore, Nox4-derived ROS can induce fibrosis via TGF-β1 activation and fibroblast differentiation into myofibroblasts. The increased apoptosis and adipogenesis in ACM patients is likely caused by co-activation of PPARγ and PPARα, which points toward an altered metabolic state of the ACM heart.

## Discussion

In ACM patients, malignant ventricular arrhythmias and SCD largely occur in the pre-clinical phase of the disease, before overt structural changes. To prevent or interfere with the disease progression, more insight into the mechanisms related to electrical instability in ACM hearts is needed. At present, a large extent of research is focused on the link between cardiac arrhythmias and metabolic disease. Mitochondrial dysfunction can affect the electrical balance in cardiomyocytes and favor arrhythmogenesis. The role of mitochondrial dysfunction in ACM pathology is still unclear, and mitochondrial biology and structure in the ACM heart remains understudied. Here, we reviewed cardiac mitochondrial dysfunction, cardiac arrhythmias and the potential links to typical hallmarks of ACM.

Mitochondrial dysfunction reduces ATP production and increases ROS production in the heart. Upon insufficient ATP supply, the predominantly closed sarcKATP channels can become conductive. Open sarcKATP channels can impair electrical conduction in the heart, by reducing cardiomyocyte excitability. In addition, increased ROS production leads to abnormal opening of various ion channels and reduces the expression of gap junctions. Mitochondrial Ca^2+^ overload (via for example mtCx43) increases ROS production, oxidizes RyR2 and subsequently induces SR Ca^2+^ leakage. This results in repolarization abnormalities, ectopic activity, conduction defects and eventually (re-entry based) cardiac arrhythmias. ROS oxidation also indirectly affects ion channels, transcription factors and (pathogenic) intracellular processes, through the multifunctional protein CaMKII. Appearance of fibrosis, apoptosis, and lipogenesis is highly related to mitochondrial (dys)function. Fibrosis formation can be induced via Cx43 hemichannel-mediated ATP release, SR Ca^2+^-driven Pannexin-1 activation and ROS-TGF-β1-Nox4 activated fibroblast differentiation. Whether some or all of these changes are present in cardiomyocytes of hearts deficient in desmosomal proteins remains to be determined. PPARγ and PPARα co-activation is a possible link between metabolic changes in PKP2-deficient patients, apoptosis and lipogenesis. Yet their relative contribution remains a subject of further investigation.

The limited amount of experimental evidence in ACM models makes it hard to determine whether mitochondrial dysfunction indeed precedes and/or accompanies ACM pathogenesis. Mutations in mitochondrial DNA can provoke mitochondrial dysfunction and activate the mechanisms we discussed. On the contrary, loss of ID integrity might also affect the intracellular ion homeostasis (e.g., Ca^2+^) and alters mitochondrial activity and homeostasis. The cardiac stress responses upon mutations in ID-associated proteins likely activates several mechanisms related to mitochondrial dysfunction, such as inflammation and apoptosis. Postulated hypotheses are largely based upon findings in experimental PKP2 models, which are of course not representative for the entire ACM population, therefore extrapolations should be done with caution. Nevertheless, these models can be very useful in discovering ACM-related mitochondrial biology and testing of therapeutic targets, as for example anti-oxidant treatment or genetic repair.

## Author Contributions

CO and LB prepared the primary manuscript. MD and TV critically revised the manuscript. CO produced the figures.

## Conflict of Interest

The authors declare that the research was conducted in the absence of any commercial or financial relationships that could be construed as a potential conflict of interest.
